# Neurofilament Light Chain: Blood Biomarker of Neonatal Neuronal Injury

**DOI:** 10.3389/fneur.2018.00984

**Published:** 2018-11-20

**Authors:** Antoinette Depoorter, Roland P. Neumann, Christian Barro, Urs Fisch, Peter Weber, Jens Kuhle, Sven Wellmann

**Affiliations:** ^1^Department of Neuropediatrics and Developmental Medicine, University Children's Hospital Basel, University of Basel, Basel, Switzerland; ^2^Department of Neonatology, University Children's Hospital Basel, University of Basel, Basel, Switzerland; ^3^Neurologic Clinic and Policlinic, Departments of Medicine, Biomedicine and Clinical Research, University Hospital Basel, University of Basel, Basel, Switzerland

**Keywords:** cerebral injury, neuropathology, biomarker, infant, parturition, prematurity

## Abstract

**Background:** Neurofilament light chain (NfL) is a highly promising biomarker of neuroaxonal injury that has mainly been studied in adult neurodegenerative disease. Its involvement in neonatal disease remains largely unknown. Our aim was to establish NfL plasma concentrations in preterm and term infants in the first week of life.

**Methods:** Plasma NfL was measured by single molecule array immunoassay in two neonatal cohorts: cohort 1 contained 203 term and preterm infants, median gestational age (GA) 37.9 weeks (interquartile range [IQR] 31.9–39.4), in whom venous and arterial umbilical cord blood was sampled at birth and venous blood at day of life (DOL) 3; cohort 2 contained 98 preterm infants, median GA 29.3 weeks (IQR 26.9–30.6), in whom venous blood was sampled at DOL 7.

**Results:** Median NfL concentrations in venous blood increased significantly from birth (18.2 pg/mL [IQR 12.8–30.8, cohort 1]) to DOL 3 (50.9 pg/mL [41.3–100, cohort 1]) and DOL 7 (126 pg/mL [78.8–225, cohort 2]) (*p* < 0.001). In both cohorts NfL correlated inversely with birth weight (BW, Spearman's rho −0.403, *p* < 0.001, cohort 1; R −0.525, *p* < 0.001, cohort 2) and GA (R −0.271, *p* < 0.001, cohort 1; R −0.487, *p* < 0.001, cohort 2). Additional significant correlations were found for maternal age at delivery, preeclampsia, delivery mode, 5-min Apgar, duration of oxygen supplementation, sepsis, and brain damage (intraventricular hemorrhage or periventricular leukomalacia). Multivariable logistic regression analysis identified the independent predictors of NfL in cohort 1 as BW (beta = −0.297, *p* = 0.003), delivery mode (beta = 0.237, *p* = 0.001) and preeclampsia (beta = 0.183, *p* = 0.022) and in cohort 2 as BW (beta = −0.385, *p* = 0.001) and brain damage (beta = 0.222, *p* = 0.015).

**Conclusion:** Neonatal NfL levels correlate inversely with maturity and BW, increase during the first days of life, and relate to brain injury factors such as intraventricular hemorrhage and periventricular leukomalacia, and also to vaginal delivery.

## Introduction

As direct access to the central nervous system (CNS) is almost impossible, neuronal biomarkers have been investigated for decades in order to improve early diagnostics, monitor disease progression and optimize care. Neurofilaments (Nf) are highly specific major neuronal scaffolding proteins comprising 4 subunits: the triplet of Nf light chain (NfL), Nf medium chain, and Nf heavy chain (NfH), and α-internexin in the CNS, or peripherin in the peripheral nervous system (1). Acute or chronic neuronal damage, including traumatic brain injury, stroke, dementia and multiple sclerosis, releases Nf fragments into the cerebrospinal fluid and eventually the blood compartment (2–6). Recent advances using highly sensitive single molecule array (Simoa) immunoassay have improved NfL detection, particularly in peripheral blood, making it a promising and readily accessible biomarker for neuroaxonal injury (7).

Whereas, circulating Nf has been extensively characterized in adults and older children with neurologic disease, data in infants and particularly newborns are sparse. One study reported raised serum NfH in children older than 6 months with febrile seizures lasting >30 min, suggesting that prolonged seizures cause some degree of neuronal damage (8). Plasma NfH in newborns with hypoxic-ischemic encephalopathy (HIE) was also higher than in healthy neonates (9, 10). Moreover, NfL levels in infants undergoing therapeutic hypothermia for HIE were significantly higher in those with unfavorable vs. favorable brain magnetic resonance imaging (MRI) outcome (11). As for mode of delivery, serum NfH levels at day of life (DOL) 2 in a small cohort of newborns did not differ between those born vaginally and those born by cesarean section (12).

Given the potential of Nf in adults with acute or chronic CNS damage and promising results in infants with HIE, we aimed to measure NfL levels by Simoa in two cohorts of preterm and term neonates in umbilical cord blood at birth and in venous blood a few days after birth.

## Materials and methods

### Study participants

The study was based on data and blood samples prospectively collected from two neonatal cohorts. Cohort 1 comprised data and blood samples from 203 preterm and term neonates, median gestational age (GA) 37.9 weeks (interquartile range [IQR] 31.9–39.4), born and cared for at the University Hospitals of Zurich and Basel, Switzerland. More specifically, it comprised 89 preterm infants (GA < 37 weeks), including 52 with GA < 32 weeks, and 114 term infants (GA ≥ 37 weeks). The study was approved by the institutional review boards of both university hospitals (Ethikkommission beider Basel, EKBB07/09, Kantonale Ethikkommission Zurich, KEK08/09). Cohort 2 comprised data and blood samples from 98 very preterm neonates (GA < 32 weeks), median GA 29.3 weeks (IQR 26.9–30.6), born and cared for at the University Hospital of Basel, Switzerland. The study was approved by the institutional review board (Ethikkommission beider Basel, EK233/13) and was carried out in accordance with the declaration of Helsinki. Written informed consent was obtained from the parents prior to enrollment.

### Clinical characteristics (Table 1)

Details of pregnancy (presence/absence of preeclampsia, amniotic infection, preterm labor, maternal age, premature rupture of membranes), delivery (umbilical artery pH, delivery modality), birth (GA, BW, sex, 5- and 10-min Apgar scores), and postnatal course to discharge home (presence/absence of sepsis and/or necrotizing enterocolitis, ultrasound brain damage with periventricular intraventricular hemorrhage [PIVH] or periventricular leukomalacia [PVL], duration of oxygen) were collected from the charts. Definitions of clinical characteristics, including preeclampsia, clinical chorioamnionitis, PIVH, and PVL, have been described previously (13), based on standardized definitions of the Swiss Neonatal Network.

**Table 1:** Descriptive statistics.

	**Cohort 1** ***n*** = **203**		**Cohort 2 *n* = 98**	
	**Moderate Preterm and Term (≥32 weeks GA) *n* = 151**	**Very preterm (< 32 weeks GA) *n* = 52**	**Very preterm (< 32 weeks GA) *n* = 98**
**NEONATAL CHARACTERISTICS**			
GA (weeks)	38.3 (37.0–40.0)	30.1 (28.3–31.3)	29.3 (26.9–30.6)
BW (g)	3270 (2710–3630)	1360 (1063–1463)	1145 (788–1413)
Sex (male, %)	87 (57.6)	25 (48.1)	52 (53.1)
Brain damage (%)	1 (0.7)	10 (19.2)	12 (12.2)
O_2_ duration (days)	0	4 (1–15.8)	2.38 (0.05–22.8)
pH umbilical artery	7.30 (7.26–7.33)	7.32 (7.29–7.37)	7.32 (7.28–7.36)
NEC (%)	0	0	3 (3.1)
Sepsis (%)	0	11 (21.2)	13 (13.3)
5-min Apgar	9 (9–9)	7 (5.25–8)	7 (6–8)
Death (%)	0	6 (11.5)	2 (2.0)
**MATERNAL CHARACTERISTICS**			
Age (years)	32 (29–36)	33 (28.3–36.0)	33 (29–36)
Amniotic infection (%)	5 (3.3)	13(25)	20 (20.4)
Preeclampsia (%)	16 (10.6)	20 (38.5)	16 (16.3)
PROM (%)	14 (9.3)	14 (26.9)	28 (28.6)
DM (%):			
Primary CS	76 (50.3)	26 (50)	27 (27.6)
Secondary CS	29 (19.2)	21 (40.4)	59 (60.2)
VD	46 (30.5)	5 (9.6)	12 (12.2)

*GA, gestational age; BW, birth weight; BD, brain damage (PIVH and/or PVL); NEC, necrotizing enterocolitis; PROM, premature rupture of membranes; DM, delivery mode; CS, cesarean section; VD, vaginal delivery. GA, BW, O_2_ duration, Apgar, pH and maternal age are presented as median and interquartile range*.

### Sample preparation and assessment of NfL

In cohort 1, venous blood (0.5 mL) was collected from the umbilical cord at birth (*n* = 185) and simultaneously with mandatory neonatal metabolic screening at DOL 3 (*n* = 39); 68 paired umbilical arterial samples were also collected at birth. In cohort 2, venous blood was collected with diagnostic blood samples at DOL 7 (*n* = 98). All samples were handled according to standard operating procedures for blood sampling in EDTA tubes, subsequent sample transfer to the central laboratory service, centrifugation, preparation of aliquots, and storage at −80°C until batch-wise analysis as described previously (14). Assay technicians were blinded to clinical information and pregnancy outcome.

NfL levels were measured by Simoa immunoassay using capture monoclonal antibody (mAB) 47:3 and biotinylated detector mAB 2:1 (UmanDiagnostics, Umea, Sweden), as previously described (15). Calibrators (neat) and serum samples (1:4 dilution) were measured in duplicate. Bovine lyophilized NfL was obtained from UmanDiagnostics. Calibrators ranged from 0 to 2,000 pg/mL. Batch-prepared calibrators were stored at −80°C. Intra- and interassay variabilities were < 10%; the few samples with intra-assay coefficients of variation >20% were remeasured.

### Data analysis

Statistical analyses were performed using SPSS for Windows version 24 (IBM) and included descriptive statistics, Spearman's rank-order correlation analyses and multiple linear regressions (MLR) using NfL as dependent variable. NfL variables were log10 transformed for the correlations and MLR. The independent variables included for MLR were based on significant correlations and significant non-parametric univariate analyses such as the Mann-Whitney U (2 levels) and Kruskal-Wallis tests (>2 levels). For cohort 1 these variables were: BW, 5-min Apgar, delivery mode (3 levels), preeclampsia, sepsis, and oxygen duration. For cohort 2 they were: BW, 5-min Apgar, sex, brain damage, sepsis, amniotic infection, and oxygen duration. Due to collinearity between BW and GA, we used only BW in MLR, where it showed stronger correlation with NfL than GA.

## Results

### Baseline NfL levels

In cohort 1 overall median venous NfL concentrations were 18.2 pg/mL (IQR 12.8–30.8) at birth and 50.9 pg/mL (41.3–100.1) at DOL 3; in cohort 2 they were 128.5 pg/mL (78.8–224.8) at DOL 7.

We split cohort 1 into a very preterm group (GA < 32 weeks; *n* = 52) and a moderate preterm and term (MPT) group (GA ≥32 weeks; *n* = 151) with fewer prematurity complications (*n* = 1 in our sample). This also enabled us to compare the first group with cohort 2. NfL levels were significantly higher in very preterm infants than in the MPT group at birth (median 32.5 pg/mL, *n* = 47 vs. 15.3 pg/mL, *n* = 138; *p* < 0.001), but not at DOL 3 (median 48.5 pg/mL, *n* = 16 vs. 51.4 pg/mL, *n* = 23; *p* = 0.668). Moreover, levels increased significantly from birth to DOL 3 in both the very preterm and MPT groups (median 32.5 vs. 48.5 pg/mL, *p* = 0.002; and median 15.3 vs. 51.4 pg/mL, *p* < 0.001), and from DOL 3 to DOL 7 in the very preterm group (median 48.5 vs. 128.5 pg/mL, *p* = 0.001) (Table 2). This increase was confirmed in cohort 1 when comparing paired samples from same infants (MPT group *n* = 16, very preterm group *n* = 11) at birth and DOL 3 (median 18.2 pg/mL vs. 49.4 pg/mL). Out of these, only in 2 very preterm infants NfL levels remained unchanged, in all other infants they increased from birth until DOL 3. Paired umbilical cord arterial and venous plasma were closely related (*R* = 0.875, *p* < 0.001). Given this close correlation and the greater number of subjects (*n* = 185), we performed all further analyses using the venous blood samples collected at birth.

**Table 2:** Cohort neurofilament light chain concentrations at birth and at days of life (DOL) 3 and 7.

**Cohort**	**Neurofilament light chain concentrations (pg/mL)**			
	**Birth (arterial)**	**Birth (venous)**	**DOL 3 (venous)**	**DOL 7 (venous)**
1: Very preterm group (GA < 32 weeks) *n* = 52		32.5 (17.6–52.5) *n* = 47	48.5 (37.6–138) *n* = 16
1: Moderate Preterm and Term group (GA ≥32 weeks) *n* = 151	17.7 (12.4–25.4) *n* = 68	15.3 (12.2–23.9) *n* = 138	51.4 (41.4–86.4) *n* = 23
2: Very preterm group (GA < 32 weeks) *n* = 98				126 (78.8–225) *n* = 98

*Median and interquartile range. GA, gestational age*.

### NfL and perinatal characteristics in cohort 1

**Figure 1:**
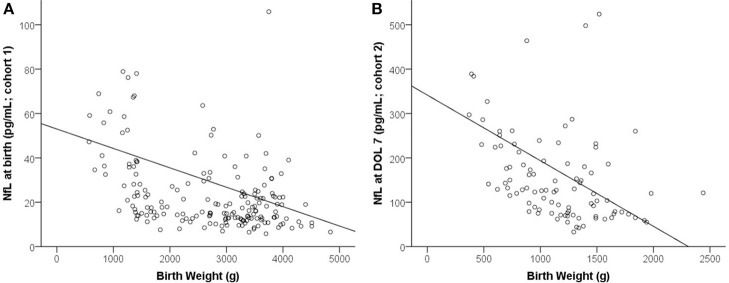
Correlation between birth weight and NfL. **(A)** Birth (cohort 1). **(B)** DOL 7 (cohort 2).

Venous cord blood at birth correlated negatively with BW (*R* = −0.403, *p* < 0.001, Figure 1), GA (*R* = −0.271, *p* < 0.001), 5-min Apgar (*R* = −0.295, *p* < 0.001), and 10-min Apgar (*R* = −0.363, *p* < 0.001). In contrast, levels correlated positively with oxygen duration (*R* = 0.333, *p* < 0.001) and delivery mode (*R* = 0.156, p = 0.034).

Presence of preeclampsia (31.0 pg/mL vs. 16.2, *p* < 0.001) and sepsis (32.6 pg/mL vs. 17.85, *p* = 0.033) were associated with higher NfL levels.

**Figure 2:**
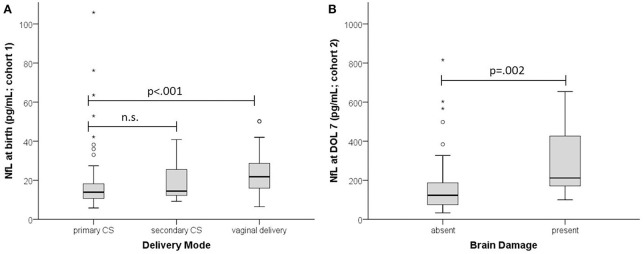
Effect of delivery mode and brain damage on NfL. **(A)** NfL at birth in infants with GA ≥ 32 weeks (cohort 1). **(B)** NfL at DOL 7 (cohort 2). Absent, no brain damage; present, PIVH and/or PVL. Boxplots are presented with median and IQR. The * are extreme outliers.

In the MPT group NfL levels at birth were significantly higher in infants delivered vaginally than by primary or secondary cesarean section (21.8 vs. 13.9 and 14.4 pg/mL; *p* = 0.002) (Figure 2). This was not the case in the very preterm group, presumably due to the few vaginal deliveries (*n* = 5 vs. *n* = 47 cesarean sections). At DOL 3 there was no significant difference (*p* = 0.07) in NfL levels between birth modalities except for vaginal delivery vs. cesarean section (110 pg/mL, *n* = 8 vs. 48.7 pg/mL, *n* = 31; *p* = 0.031).

MLR testing for the best independent predictors of NfL levels at birth used BW, 5-min Apgar, delivery mode, preeclampsia, sepsis and oxygen duration as explanatory variables. The model was significant (*F*_(6, 176)_ = 8.655, *p* < 0.001), explaining around 23% of NfL variance (R^2^ = 0.228). The predictors were BW (beta = −0.297, *p* = 0.003), delivery mode (beta = 0.237, *p* = 0.001), and preeclampsia (beta = 0.183, *p* = 0.022).

### NfL and perinatal characteristics in cohort 2

NfL at DOL 7 correlated negatively with the main neonatal characteristics such as BW (*R* = −0.525, *p* < 0.001, Figure 1), GA (*R* = −0.487, *p* < 0.001), and 5- and 10-min Apgar (*R* = −0.247, *p* = 0.014; *R* = −0.228, *p* = 0.024). Correlation was positive with oxygen duration (*R* = 0.358, *p* < 0.001) and maternal age (*R* = 0.353, *p* < 0.001).

Brain damage (211.5 pg/mL vs. 123, *p* = 0.002) and sepsis (184 pg/mL vs. 124.5, *p* = 0.020) were associated with higher NfL levels. Delivery mode had no significant impact (*p* = 0.624).

MLR analysis of cohort 2 used BW, 5-min Apgar, sex, brain damage, sepsis, amniotic infection, and oxygen duration as explanatory variables. The regression model explained around 37% of NfL variance (*R*^2^ = 0.366, *F*_(7, 89)_ = 7.331, *p* < 0.001). Only BW (beta = −0.385, *p* = 0.001) and brain damage (beta = 0.222, *p* = 0.015) contributed significantly to predicting NfL (Figure 2).

## Discussion

Neuronal injury marker NfL has proved a sensitive and specific biomarker in adult peripheral blood, serving as a promising adjunct to monitoring and decision-making in acute and chronic neurologic disease (16, 17). Our study provides a first insight into neonatal NfL levels in term and preterm infants. The major findings are that NfL levels increase over the first few days of life, relate inversely to prematurity and BW, and identify BW, delivery mode, preeclampsia and brain damage as independent predictors.

NfL levels at birth in MPT infants resemble those in healthy adults (15). By DOL 3 they rise to the levels seen in adults with neurodegenerative disease such as multiple sclerosis (15). At DOL 7 in very preterm infants NfL levels are in the range of asphyxiated neonates at DOL 4 (11).

The main influencers of NfL in both cohorts were BW and maturity: birth and neonatal levels were both higher in low BW infants (Figure 1), perhaps because brain vulnerability to neuronal injury increases with prematurity. Alternatively, high NfL levels in preterm infants might be due to high neuronal turnover in general, with the much higher postnatal levels at DOL 3 and DOL 7 (Figure 2) simply reflecting a neuronal stress reaction to birth, as in healthy term neonates.

Preterm infants are at risk for perinatal brain damage, in particular PIVH and PVL (18). In our sample those with evidence of brain damage had significantly higher NfL levels than those without (Figure 2). Brain damage leads directly to neuronal injury, to a degree objectifiable by NfL: levels are higher in asphyxiated neonates with unfavorable brain MRI outcome (11). As in adults, cerebrovascular accident results in immediately higher NfL levels (19), compared to the more gradual neuronal damage seen in neurodegenerative disease (20).

In addition to a direct effect of brain damage, we identified two other stressors that increase NfL, namely delivery mode and preeclampsia. Levels were higher in infants delivered vaginally than by cesarean section (Figure 2), suggesting greater neuronal injury and confirming vaginal delivery as one of life's strongest stressors, causing incommensurable release of various fetal stress hormones (21). Preeclampsia, a pregnancy-specific syndrome defined by high blood pressure and other morbidities (22), was the additional stressor, raising NfL levels at birth even after adjustment for BW and GA. Our finding is consistent with the recent report of raised NfL levels in women with preeclampsia (23). Maternal hypertension is closely linked to placental insufficiency which compromises fetal perfusion and may cause cardiovascular disease later in life (24). Our data indicate that preeclampsia involves a risk of neuronal damage in the unborn child.

While the main source of NfL is considered to be the central nervous system, peripheral damage may contribute to increased NfL values as well, as recently revealed by studies on peripheral neuropathies (25, 26). Increased blood levels of the muscle enzyme creatine kinase in newborn infants after vaginal deliveries compared to cesarean sections have been reported (27). They support the notion that increased NfL in these babies may result, at least in part, from peripheral neuronal damage. However, data on the central nervous system biomarker S100 B measured in the maternal serum and cord blood show clearly increased S100B values after vaginal delivery compared to cesarean section (28). It has been shown previously that extracranial sources of S100B do not affect serum levels (29). Taken together, the findings of Schulpis KH et al. corroborate our data that increased levels of the neuronal injury markers S100B and NfL might be caused by the compression on the fetus' brain during delivery.

Further, S100B levels in neonates with HIE exceeded those in healthy controls, proportionately to disease severity and worse outcome (30). Although S100B levels decreased overall from DOL 1 through DOL 9 (31), levels in preterm and term neonatal saliva followed a pattern similar to NfL, being higher in preterm than in term infants and correlating negatively with GA (32). Nerve growth factor (NGF) is a neurotrophic factor involved in brain development and neuroplasticity following brain damage. Unlike NfL, NGF levels in maternal and cord plasma are lower in preterm than in term deliveries (33).

To date the metabolism of NfL in cerebrospinal fluid (CSF) and blood is largely unknown, ways of elimination or protein degradation have not been described. One study examined the influence of blood brain barrier permeability and blood NfL levels. In this study there was no correlation between serum NfL concentration and CSF/serum albumin ratio (34).

Study limitations include the relatively few subjects sampled at DOL 3, which may account for the non-significant difference between very preterm and MPT infants at DOL 3. In the first week of life there is an apparent increase in NfL levels, but in the absence of data points post-DOL 7, the subsequent profile of NfL requires elucidation in further studies. Nor can we exclude other confounders that might influence and explain NfL. Cognitive outcome studies will need to confirm the use of NfL as a predictive biomarker of brain damage and eventual neurodevelopmental deficit. Such early biomarkers are sorely needed to complement ultrasound or MRI in conditions such as PVL (18). In addition, future studies may explore NfL together with other potentially promising biomarkers of brain damage (35). More generally, research is required to explore and disentangle the causes of the high degree of neuronal injury in the preterm brain.

## Conclusion

This study provides an initial insight into neuronal injury marker NfL in term and preterm infants. Levels increase through the first week of life. They relate inversely to GA and BW and are higher in brain injury. Obstetric parameters such as delivery mode and preeclampsia also raise NfL levels. Our study supports the use of NfL in neonates to help us understand the factors leading to neuroaxonal injury and how we might monitor and prevent them.

## Data availability

The raw data supporting the conclusions of this manuscript will be made available by the authors, without undue reservation, to any qualified researcher.

## Author contributions

SW and UF designed the study. SW and RN collected the data. JK and CB assayed the serum samples. AD analyzed the data and wrote the manuscript together with SW and PW. All authors provided critical feedback and helped to improve the manuscript.

### Conflict of interest statement

The authors declare that the research was conducted in the absence of any commercial or financial relationships that could be construed as a potential conflict of interest.

## Abbreviations

Nf: Neurofilament
NfL: Neurofilament Light Chain
GA: Gestational Age
BW: Birth Weight
DOL: Day of Life
MPT: Moderate Preterm and Term.
